# 38460 Independent Investigator Incubator (I3) yields external funding within three years for the majority of junior faculty

**DOI:** 10.1017/cts.2021.567

**Published:** 2021-03-30

**Authors:** John Paul Spence, Christina R. Santangelo, Jennifer L. Buddenbaum, Aaron E. Carroll, Matthew R. Allen

**Affiliations:** Indiana University School of Medicine

## Abstract

ABSTRACT IMPACT: The Independent Investigator Incubator program provides 1:1 mentoring from ‘super-mentors’ to enhance junior faculty careers in research. OBJECTIVES/GOALS: In 2014, the Indiana University School of Medicine (IUSM) in collaboration with the Indiana CTSI established the Independent Investigator Incubator (I3) Program. The I3 Program is designed to provide 1:1 mentoring for new research faculty during the crucial early years of their careers. Our goal is to provide an overview of the I3 design and 5-year data. METHODS/STUDY POPULATION: The I3 Program employs a resource-sharing, centralized design that provides concentrated 1:1 mentorship from a senior faculty ‘super mentor’ as well as other resources, such as grant writing support. Unlike many mentorship programs, I3 mentors closely interact with the mentees within the School and are compensated for their efforts (5% full-time equivalency per mentee, max of 15%). The number of ‘super mentors’ has grown from 6 to 15 faculty over 5 years, and mentors typically serve 4 to 5 mentees. Mentee applications are accepted on a rolling enrollment basis. The I3 mentees represent a diverse group based on sex, ethnicity, terminal degree, academic track, and discipline. Mentors and mentees have annual reviews through the program. RESULTS/ANTICIPATED RESULTS: In five years, 110 mentees have enrolled in the I3 program. Upon entering, 53% had no external funding, 28% had internal funding, 12% had K-awards, 7% had R03/R21 awards. Over the first five years, 75% have received extramural funding. The median funding was $340,000 with nearly a third of mentees securing grants > 1 million in direct costs. For mentees who joined the program in its first three years (n=59), the average time to a notable extramural grant (defined as a NIH or foundation grant >$300K direct costs) was 2.2 years (median - 2.6 years). Nearly all mentees were satisfied with their mentor pairing based on the mentor’s ‘availability’ and ‘valuable feedback,’ and all mentees wanted the mentoring relationship to continue DISCUSSION/SIGNIFICANCE OF FINDINGS: Since 2014, the I3 Program has had a positive impact on the careers of junior faculty at IUSM as determined by faculty satisfaction and funding metrics. Future focus areas will include developing criteria/models for graduating from the program to balance fiscal sustainability with mentee needs during their transition to mid-career.

